# A Visual Dual-Aptamer Logic Gate for Sensitive Discrimination of Prion Diseases-Associated Isoform with Reusable Magnetic Microparticles and Fluorescence Quantum Dots

**DOI:** 10.1371/journal.pone.0053935

**Published:** 2013-02-05

**Authors:** Sai Jin Xiao, Ping Ping Hu, Li Qiang Chen, Shu Jun Zhen, Li Peng, Yuan Fang Li, Cheng Zhi Huang

**Affiliations:** 1 Education Ministry Key Laboratory on Luminescence and Real-Time Analysis, College of Chemistry and Chemical Engineering, Southwest University, Chongqing, China; 2 Jiangxi Key Laboratory of Mass Spectrometry and Instrumentation, Department of Applied Chemistry, East China Institute of Technology, Nanchang, Jiangxi Province, China; 3 College of Life Science, Southwest University, Chongqing, China; 4 College of Pharmaceutical Sciences, Southwest University, Chongqing, China; The Scripps Research Institute Scripps Florida, United States of America

## Abstract

Molecular logic gates, which have attracted increasing research interest and are crucial for the development of molecular-scale computers, simplify the results of measurements and detections, leaving the diagnosis of disease either “yes” or “no”. Prion diseases are a group of fatal neurodegenerative disorders that happen in human and animals. The main problem with a diagnosis of prion diseases is how to sensitively and selectively discriminate and detection of the minute amount of PrP^Res^ in biological samples. Our previous work had demonstrated that dual-aptamer strategy could achieve highly sensitive and selective discrimination and detection of prion protein (cellular prion protein, PrP^C^, and the diseases associated isoform, PrP^Res^) in serum and brain. Inspired by the advantages of molecular logic gate, we further conceived a new concept for dual-aptamer logic gate that responds to two chemical input signals (PrP^C^ or PrP^Res^ and Gdn-HCl) and generates a change in fluorescence intensity as the output signal. It was found that PrP^Res^ performs the “OR” logic operation while PrP^C^ performs “XOR” logic operation when they get through the gate consisted of aptamer modified reusable magnetic microparticles (MMPs-Apt1) and quantum dots (QDs-Apt2). The dual-aptamer logic gate simplifies the discrimination results of PrP^Res^, leaving the detection of PrP^Res^ either “yes” or “no”. The development of OR logic gate based on dual-aptamer strategy and two chemical input signals (PrP^Res^ and Gdn-HCl) is an important step toward the design of prion diseases diagnosis and therapy systems.

## Introduction

Logic gate, an original concept of a programmable computer, performs a logical operation on one or more logic inputs and produces a single logic output. For example, the OR logic gate combines input 1 (*i*_1_) and input 2 (*i*_2_), and results in the output of 1 when *i*_1_ and/or *i*_2_ is 1, while XOR logic gate is a combinatorial logic gates with the output of 1 when *i*_1_ is not equal to *i*_2_. By analogy, molecular logic gates, which have attracted increasing research interest, perform logical operation inputs resulting from chemical or biological processes and generate a single logic output such as spectral or electrochemical signal. Several prototypes on the basis of DNA [1]–[2], RNA [3], and deoxyribozymes [4], biochemical pathways in living cell and organic molecules [5]–[7] have been designed up to now. It can be seen that the molecular logical gates simplify the results of measurements and detections, leaving the diagnosis of disease either “yes” or “no”, or the detection of analytes in samples either “have” or “none”.

Prion diseases, a group of fatal neurodegenerative disorders, happens in human and animals. The emergence of vCJD and its linkages to BSE triggers the researchers to develop methods for prion diseases diagnosis at the pre-symptomatic stage [8], [9]. As the pathological form of prion protein, PrP^Res^, is abundant only at the late stages in brain, the main problem for prion diseases diagnosis at pre-symptomatic stage is how to detect the minute quantities of PrP^Res^ in complex biological systems. Early methods such as western blot assays and enzyme-linked immunosorbent assays (ELISAs) are insuficient sensitive. Thus researchers attempted to use conformational antibodies with various spectroscopic techniques to improve the sensitivity [10]–[13], however, the worldwide application of such methods is limited by the usefulness and specificity of antibodies or expensive and sophisticated equipment.

With the purpose of addressing the limitations mentioned above, we developed a sensitive and selective PrP^Res^ discrimination and detection assay taking the advantages of aptamers since more and more aptamers against prion protein were selected based on the known capacity of PrP to bind nucleic acids [14]–[16]. Aptamers have been applied in protein detection [17], [18], diseases diagnosis [19], [20], cell imaging and tracking [21], [22] ect. owing to the advantages of exceptional stability, easy manipulation and reproducibility, non-toxic and diagnostic potential. Previous aptamer-based assays mainly adopted single aptamer strategy, however the sensitivity and specificity of such assay is usually insufficient for target detection especially when they applied in complex biological samples [23], [24]. Our pioneer work have also demonstrated that highly sensitive and selective discrimination and detection of PrP^Res^ in serum and brain homogenate could be achieved without sample pretreatment by dual-aptamer strategy, and the results showed that the sensitivity of dual aptamer-based assay is 1000 folds higher than that of antibody-based method [25]. Inspired by the advantages of molecular logic gate and dual-aptamer strategy, we further concieved a new concept for dual-aptamer logic gate that responds to two chemical input signals (PrP^C^ or PrP^Res^ and Gdn-HCl) and generates a change in fluorescence intensity as the output signal. It is found that PrP^Res^ performs OR logic gate operation and PrP^C^ performs XOR logic gate operation on the gate consisted of aptamers modified magnetic microparticles (MMPs-Apt1) and quantum dots (QDs-Apt2). The development of OR logic gate based on dual-aptamer strategy and two chemical input signals (PrP^Res^ and Gdn-HCl) is an important step toward the design of prion diseases diagnosis systems.

## Materials and Methods

### Materials

Two aptamers, Apt1, NH2-CTT ACG GTG GGG CAA TT, and Apt2, Bio-GTT TTG TTA CAG TTC GTT TCT TTT CCC TGT CTT GTT TTG TTG TCT, were selected by Takemura and Bibby [26], [27], respectively, and synthesized by Sangon Tech. Ltd. (Shanghai, China) without further purification. Streptavidin modified quantum dots (QDs) were purchased from Jiayuan Quantum Dot Co. Ltd. (Wuhan, China). Guanidine hydrochloride (Gdn-HCl) was commercially available from Genview (USA). Ultrapure water (18.2 MΩ, LD-50G-E Lidi Ultra Pure Waters System, Chongqing, China) was used throughout. Bovine serum albumin (BSA) and pepsin were purchased from Shanghai Biochemicals (Shanghai, China). Other commercial reagents such as sodium chloride and nickel chloride were analytical reagent grade without further purification.

### Apparatus

A Hitachi F-2500 fluorescence spectrophotometer and a U-3010 spectrophotometer (both were from Hitachi, Tokyo, Japan) were used to measure the fluorescence and absorption, respectively. A J-810 spectropolarimeter (JASCO Co., Japan) was applied to obtain the circular dichroism (CD) spectra, and a KQ-100 ultrasonic processor (Kunshan Ultrasonic Instruments Factory, Jiangsu, China) was employed for the dissociation of MMPs. The fluorescence imaging was acquired with a IX81 microscope with a 10× objective (Olympus, Japan).

### Coupling of MMPs with Apt1

To improving the immobilization of aptamer on MMPs, Fe_3_O_4_ magnetic particles were prepared according to our previous method [28] with some modifications. Firstly, Fe_3_O_4_ MMPs were prepared by co-precipitating divalent and trivalent ions in alkaline solution under hydrothermal conditions [29], [30]. Secondly, tetraethyl orthosilicate (TEOS) was used to generate silica coatings on the surface of MMPs [31]. Thirdly, NH_2_-silanization of the silica coated Fe_3_O_4_ MMPs were carried out by adding 0.5 ml of 3-aminopropyltriethoxysilane (APTES) to the solution and continuously stirring for 30 min. Then, the NH_2_-silanized Fe_3_O_4_ MMPs were washed with ethanol for 3 times and dried completely in oven for 30 min at 110°C.

For the coupling of Apt1 to MMPs, 10 mg MMPs was incubated in 0.01 mol/L PBS (pH 7.4) containing 2.5% (*v*/*v*) glutaraldehyde (GA) at 37°C for 1 hour with gently stirring, equilibrated overnight at 37°C with 1.4 nmol NH_2_-Apt1 after removed redundant GA, and then washed several times and finally re-suspended in 10 mL 0.01 mol/L PBS (pH 7.4). The coupling efficiency was determined by the decreased absorbance of Apt1 at 260 nm in aqueous medium (refer to Figure S1).

### Purification of Recombination Human Cellular Prion Protein (PrP^C^) and the Conversion of PrP^C^ to PrP^Res^

PrP^C^ was prepared following the reference of Xiao's group and our previous publications [25], [32]. Firstly, the plasmid of recombinant human prion protein (23–231) was constructed and expressed in *Escherichia coli* BL21 (DE3). For protein purification, 50 μg/mL isopropyl-d-thiogalactopyranoside was used to induce the fresh overnight culture and the cells were harvested by centrifugation after 6 hours, then sonicated in lysis buffer (50 mmol/L NaH_2_PO_4_, 300 mmol/L NaCl, and 10 mmol/L imidazole, pH 8.0), and denatured in 6 mol/L Gdn-HCl overnight, then purified by nickel-nitrilotriacetic acid agarose resin (Genview). The purified proteins were analyzed by SDS-polyacrylamide gel electrophoresis and CD spectra.

PrP^Res^ was prepared according to Bocharova *et al* [33]. In short, dilute the 73.8 μmol/L *α*-form rPrP to 22 μmol/L and incubated at 37°C for 48 hours with 3 mol/L urea, 1 mol/L Gdn-HCl, 150 mmol/L NaCl at pH 4.0 in 20 mmol/L sodium acetate buffer, then dialysis with 20 mmol/L sodium acetate buffer. The conformation of PrP^Res^ was confirmed by CD spectra (see in Ref. 25) [25].

### Discrimination and Detection of PrP^Res^

For the discrimination of PrP^Res^, the native state solutions or denatured state solutions that were pre-treating with 4 mol/L Gdn-HCl at 83°C for 10 min were incubated with 60 μL MMPs-Apt1 and 30 μL 1.0 mg/mL BSA in 0.02 mol/L PB (pH 6.1) containing 0.2 mol/L NaCl for 30 min at room temperature. After remove abundant proteins by magnetic separation, the re-suspension were incubated with 40 μL QDs-Apt2 in 0.02 mol/L PB (pH 7.4) containing 0.2 mol/L NaCl at room temperature for 60 min with end-over-end rotation. Then an external magnet was used to eliminate free QDs-Apt2. The fluorescence of Apt1-PrP-Apt2 conjugates was measured with the excitation at 365 nm and emission at 608 nm.

For PrP^Res^ detection, the samples were pretreated with 4 mol/L Gdn-HCl at 83°C for 10 min to eliminate the influence of PrP^C^, and then processed the same procedure as the discrimination of PrP^Res^.

### Reuse of MMPs-Apt1 Probe

With the purpose of reusing MMPs-Apt1, the Apt1-PrP-Apt2 conjugates were incubated in 1 mol/L NaOH at 50°C for 10 min and the regenerated MMPs-Apt1 probe were collected by an external magnet.

## Results and Discussion

### The Principle of Dual Aptamer Strategy

**Figure 1:**
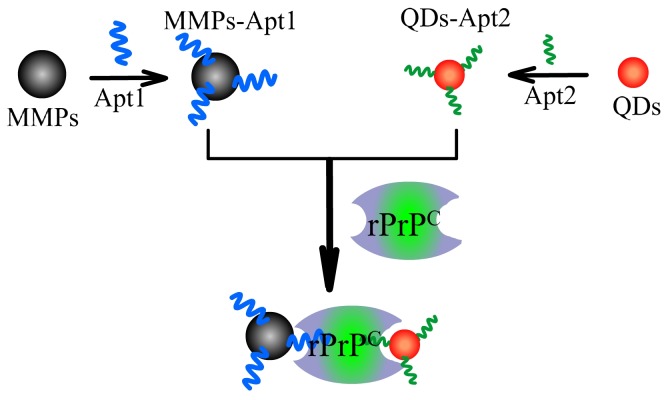
Schematic representation of dual-aptamer strategy for prion proteins detection.

**Figure 2:**
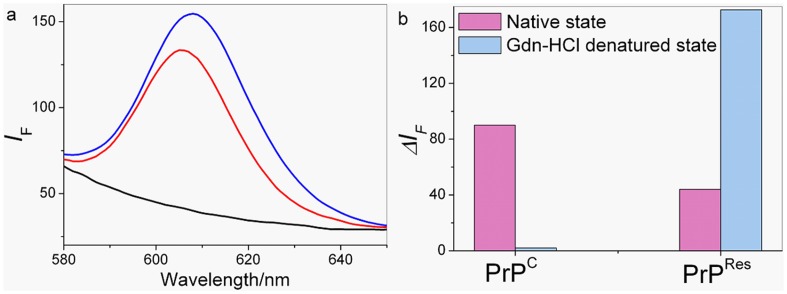
Fluorescence response of dual-aptamer strategy incubating with PrP. (a) The fluorescence spectra of MMPs-Apt1 and QDs-Apt2 incubated with 2.56×10^−6^ mol/L PrP^C^ (blue line) and 2.03×10^−6^ mol/L PrP^Res^ (red line), respectively. The black line represents control. (b) Fluorescence changes of MMPs-Apt1 and QDs-Apt2 in native state (NS) and denatured state (DS) PrP. PrP^C^ and DS-PrP^C^, 1.52×10^−6^ mol/L; PrP^Res^ and DS-PrP^Res^, 1.14×10^−6^ mol/L.

Our strategy starts with the chemical modification of MMPs and QDs with Apt1 and Apt2, respectively (Figure 1). When PrP gets through the gate made up by MMPs-Apt1 and QDs-Apt2, specific recognition of the two aptamers with the corresponding epitopes of PrP occurs, which results in the formation of fluorescent cocktails of MMPs-Apt1-PrP-Apt2-QDs. As MMPs have exceptional separation ability and QDs have high photoluminescence quality, the highly fluorescent cocktails in the aqueous mediums can be separated by the outer magnetic field. It can be seen that the fluorescence intensities got increased dramatically either PrP^C^ or PrP^Res^ was incubated with MMPs-Apt1 and QDs-Apt2 (Figure 2a), suggesting that the two aptamers are both able to bind with PrP simultaneously and the cocktails of MMPs-Apt1-PrP-Apt2-QDs formed.

### Discrimination and Detection of PrP^Res^

**Figure 3:**
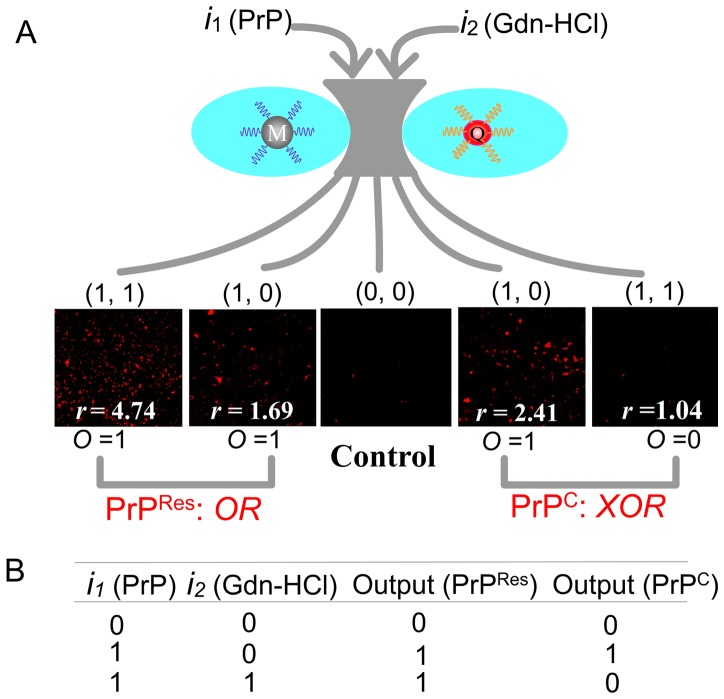
Schematic presentation (a) and the truth table (b) of dual-aptamer based logic gate. Input of *i*_1_ (PrP^C^ or PrP^Res^) for the formation of the cocktail of MMPs-Apt1-PrP-Apt2-QDs lead to an enhanced fluorescence emission (output = 1), and the input of *i*_2_ (Gdn-HCl) leads to the fluorescence different. For PrP^Res^, the fluorescence of the cocktail gets increased dramatically (output = 1), behaving similar to the OR logic gate, while for PrP^C^, the fluorescence gets disappeared (output = 0), behaving similar to the XOR logic gate. *r* represents the fluorescence ratios of (*i*_1_ = 1, *i*_2_ = 0) or (*i*_1_ = 1, *i*_2_ = 1) condition to (*i*_1_ = 0, *i*_2_ = 0) condition.

It has been reported that the resistance of PrP^C^ and PrP^Res^ to denaturing detergents is different, PrP^C^ is sensitive to denaturing detergent while PrP^Res^ is partly resistant. When treated with denaturing detergent, the structure of PrP^C^ is destroyed while the binding epitope of 90–231 of PrP^Res^ become more accessible [34]. According to the references, the binding epitopes of Apt 1 and Apt2 correspond to the 23–90 and 90–231 amino acids of PrP, respectively [26], [27]. On the basis of these, it can be assumed that PrP^Res^ could be discriminated from PrP^C^ based on the different affinities of the two aptamers to DS-PrP. As shown in Figure 2b, MMPs-Apt1 and QDs-Apt2 bind with NS-PrP^C^ or NS-PrP^Res^ simultaneously with the result of forming the cocktails of MMPs-Apt1-PrP-Apt2-QDs since the two binding epitopes of native state PrP^C^ (NS-PrP^C^) and native state PrP^Res^ (NS-PrP^Res^) are intact, and thus high fluorescence signals could be observed easily. However, when denatured PrP (DS-PrP^C^ or DS-PrP^Res^) were incubated with MMPs-Apt1 and QDs-Apt2, distinct fluorescence responses could be obtained for the reason that PrP^C^ and PrP^Res^ display different detergent denaturation curves [35], [36]. PrP^C^ is sensitive to denaturing detergent and the epitope of DS-PrP^C^ for Apt1 was destroyed when PrP^C^ was pre-treated with Gdn-HCl, therefore the cocktails can not formed with the result of non fluorescence observed. What is contrary, PrP^Res^ is resistant to Gdn-HCl and the binding epitope of DS-PrP^Res^ for Apt2 becomes more accessible after Gdn-HCl treatment, which results in the enhancement of fluorescence when MMPs-Apt1 and QDs-Apt2 incubated with DS-PrP^Res^. These results are consistent with that of Bibby *et al* and our previous work [25], [27], showing that guanidinium denaturation reduces the binding ability of aptamer to PrP^C^ and increases the binding ability of aptamer to PrP^Res^. The strong fluorescence emission of the cocktails of MMPs-Apt1-PrP-Apt2-QDs could also be observed by a fluorescence microscope. As seen in Figure 3a, both NS-PrP^C^ (the 2^nd^ picture) and NS-PrP^Res^ (the 4^th^ picture) display strong fluorescence intensities, and DS-PrP^C^ (the 3^rd^ picture) shows non-fluorescence emission while DS-PrP^Res^ (the 1^st^ picture) displays enhanced fluorescence compared with that of NS-PrP^Res^. The same resistance of PrP^Res^ to Gdn-HCl is similar to the results founded in the reported conformational discrimination assays [34], [37], [38].

**Figure 4:**
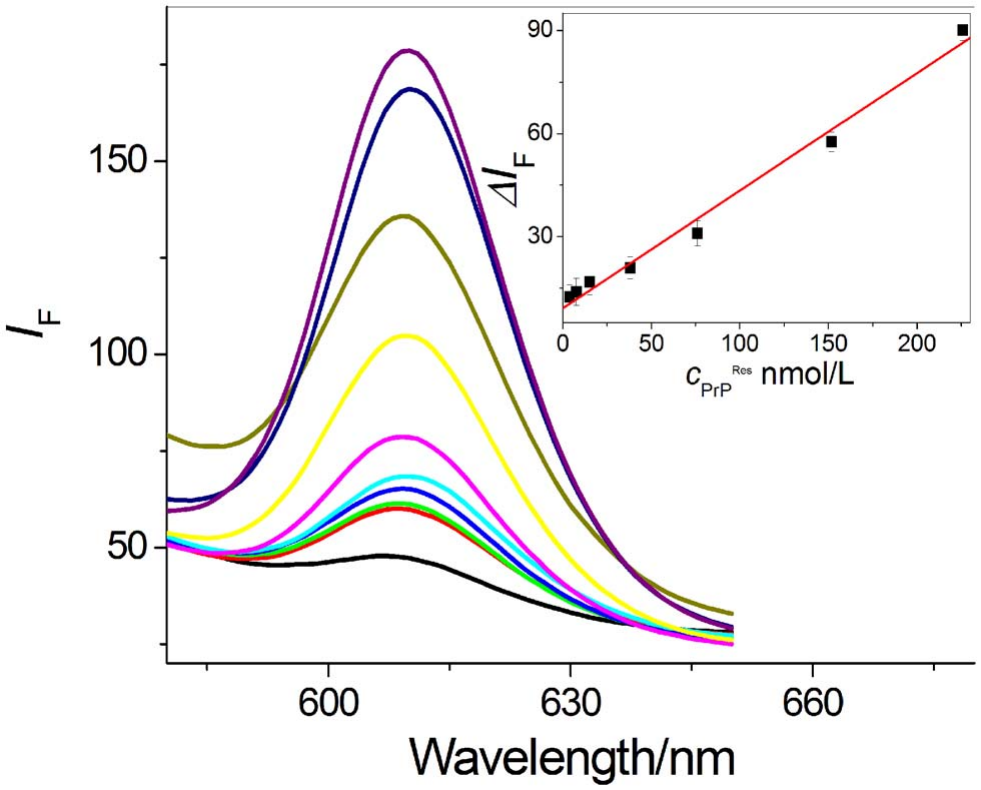
Detection of DS-PrP^Res^ with the dual-aptamer strategy. The linear equation is *ΔI*_F_ = 9.23+0.34 *c*_PrP_^Res^ in the range of 3.84–226.0 nmol/L with the correlation coefficient *R* of 0.994 (n = 7).

In order to accomplish sensitive PrP^Res^ detection, MMPs-Apt1 and QDs-Apt2 were incubated with different content of PrP^Res^. As seen in Figure 4, sequential increases of fluorescence emission were observed, and there is a good linear relationship between the enhanced fluorescence and the concentration of PrP^Res^ ranging from 3.84 to 226.0 nmol/L with the correlation coefficient of 0.994 (n = 7). It was found that the lowest concentration resulted in positive signal response with the present dual-aptamer strategy was 85.5 pmol/L, which is about 1000-folds lower than the detection limits of current Abs-mediated assays [9].

### The Construction of Dual-Aptamer Logic Gate

In such cases, a new concept of dual-aptamer logic gate can be conceived. The new dual-aptamer logic gate have two inputs, one is PrP (*i*_1_, PrP^C^ or PrP^Res^) and the other is Gdn-HCl (*i*_2_), and one fluorescence ratio (*r*) output which represents the fluorescence intensities of *i*_1_ = 1, *i*_2_ = 0 condition or *i*_1_ = 1, *i*_2_ = 1 condition to that of *i*_1_ = 0, *i*_2_ = 0 condition (*r*  =  *I*_(i1 = 1, i2 = 0) or_ *I*_(i1 = 1, i2 = 1)_ /*I*_(i1 = 0, i2 = 0)_). The output is considered to be 0 when *r* is lower than 1.10, whereas an output is interpreted as 1 when *r* is higher than 1.50. It should be noted that the two thresholds level must be applied to define a forbidden range in conventional electronic gates, and any output falling between the two values is considered as an invalid result of a logic operation [39].

As a proof-of-concept for the dual-aptamer logic gate, the fluorescence responses of PrP^Res^ can be readily exploited in the design of an OR logic gate. The fluorescence ratio output (*O*) obtained by PrP^Res^ (*i*1) and Gdn-HCl (*i*2) as inputs are shown in Figure 3a. When *i*1 and *i*2 are equal to 0 (i.e. no PrP^Res^ and Gdn-HCl, 0–0), the fluorescence ratio *r* was calculated (*r* = 1.0) and *O* = 0. When PrP^Res^ was input (1–0), the fluorescence ratio *r* was calculated (*r* = 1.69) and *O* = 1. However, if PrP^Res^ and Gdn-HCl simultaneously get through the gate made of MMPs-Apt1 and QDs-Apt2 (i.e. PrP^Res^ and Gdn-HCl, 1–1), the fluorescence significantly increased and the fluorescence ratio *r* = 4.74 (*O* = 1). The truth table matches that for the OR gate, as shown in Figure 3b. On the contrary, PrP^C^ displays different Gdn-HCl denaturation curve, as shown in Figure 2b, and thus the fluorescence responses are different from those of PrP^Res^. It can be seen in Figure 2b, the fluorescence ratio *r* = 2.41 and *O* = 1 when PrP^C^ was input (1–0). However, the fluorescence ratio *r* = 1.04 and *O* = 1 if PrP^C^ and Gdn-HCl simultaneously get through the gate (i.e. PrP^C^ and Gdn-HCl, 1–1), which is in accordance with XOR gate behavior.

### Reuse of MMPs-Apt1 Probe

**Figure 5:**
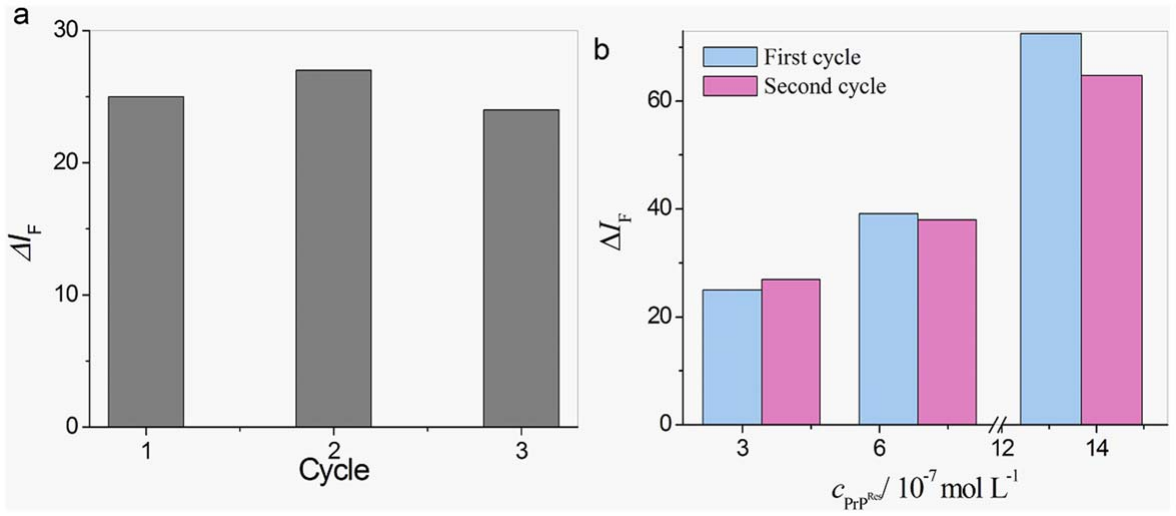
Reuse of MMPs-Apt1. (a) Good signal response could be achieved over three cycles. DS-PrP^Res^ concentrations was 3.42×10^−7^ mol/L. (b) The MMPs-Apt1 could be reused without loss of sensitivity in the second cycle when DS-PrP^Res^ are 3.42, 6.84, 13.86×10^−7^ mol/L.

It can be seen that the sensitivity of MMPs-Apt1 could be preserved even over three cycles (Figure 5a), and the results were accordance with those of our previous work [25]. The reason might be that only a surface-tethered monolayer of Apt1 was involved in the modification of MMPs surfaces. Then the reuse of MMPs-Apt1 under different PrP^Res^ concentrations were discussed, as seen in Figure 5b, the fluorescence emission intensities in the second cycle were similar to those in the first cycle, which suggest that the MMPs-Apt1 probe could be reused without loss of sensitivity in the second cycle. It can also be found that the fluorescence intensities under higher concentration in the second cycle were decreased compared with those in the first cycle, and the reasons were still being explored. The dissociation of PrP and MMPs-Apt1 could also be observed from the increasing fluorescence of the aqueous medium separated by magnetic field (Figure S2). Moreover, it was found that small peaks around 608 nm were observed from the suspension of reused MMPs-Apt1 probe (refer to Figure S3), and two possibilities might account for this phenomenon. The first one might be the incomplete dissociation of PrP from the MMPs-Apt1 surface, which can be avoided by further addition of NaOH. Since NaOH denaturation involved not only in PrP but also in the blocking agent of BSA, the nonspecific absorption of QDs-Apt2 on the surface of MMPs-Apt1 might be the other reason.

## Conclusion

In summary, OR and XOR logic gates based on two inputs (PrP and Gdn-HCl) and a fluorescence output were demonstrated taking the advantages of exceptional separation ability of MMPs, high photoluminescence quality of QDs, and the unusual sensitivity and selectivity of dual-aptamer strategy. The two aptamers provide a way for inputs and output of individual element to link each other in the dual-aptamer design. In contrast to all previously reported aptamer-based systems, the present dual-aptamer logic gate possesses the following advantages. Firstly, the dual-aptamer strategy achieved sensitive and selective PrP^Res^ discriminating, and dual-aptamer logic gate further simplifies the result of detection, leaving the detection of PrP^Res^ either “Yes” or “No”. Secondly, the inputs (PrP and Gdn-HCl) and gates (MMPs-Apt1 and QDs-Apt2) used here are all cost-saving and chemical stable molecules, which are crucial for the development of smaller, more effective molecular-scaled computers [4]. Thirdly, the MMPs-Apt1 probe could be reused without loss of sensitivity over three cycles under different concentrations. These findings should give helpful insights for the development of new dual-aptamer based device for prion diseases diagnosis at pre-symptomatic stage. To achieve this goal, further work should mainly be concentrated on the improvement of sensitivity, which might come true in virtue of the following strategies. The first one is to amplify the existing signal, for example, mass spectrometry might be used for the detection of Cd^2+^ concentration from QDs-Apt2. The other one is to concentrate the minute quantities of PrP^Res^ in samples by the exceptional separation ability of MMPs-Apt1.

## Supporting Information

Figure S1 **The absorption spectrum of Apt1 control (blank line) and the aqueous medium of MMPs-Apt1 after separated by external magnet (red line).**
(TIF)

Figure S2 **Fluorescence spectra of the supernatant of the cocktail of MMPs-Apt1-PrP-Apt2-QDs separated by magnetic field.** PrP^Res^: 0.00, 3.42, 6.84, 13.86×10^−7^ mol/L from the bottom up.
(TIF)

Figure S3 **The fluorescence spectra of reused MPs-Apt1.** The concentrations of DS-PrP^Res^ are corresponding to 0, 3.42, 6.84, 13.86×10^−7^ mol/L from the bottom up.
(TIF)
