# Supplementary figures and images for: A Visual Dual-Aptamer Logic Gate for Sensitive Discrimination of Prion Diseases-Associated Isoform with Reusable Magnetic Microparticles and Fluorescence Quantum Dots
Source: PLoS One. 2013 Feb 5;8(2):e53935. doi: 10.1371/journal.pone.0053935 (PMC3564804; doi:10.1371/journal.pone.0053935)

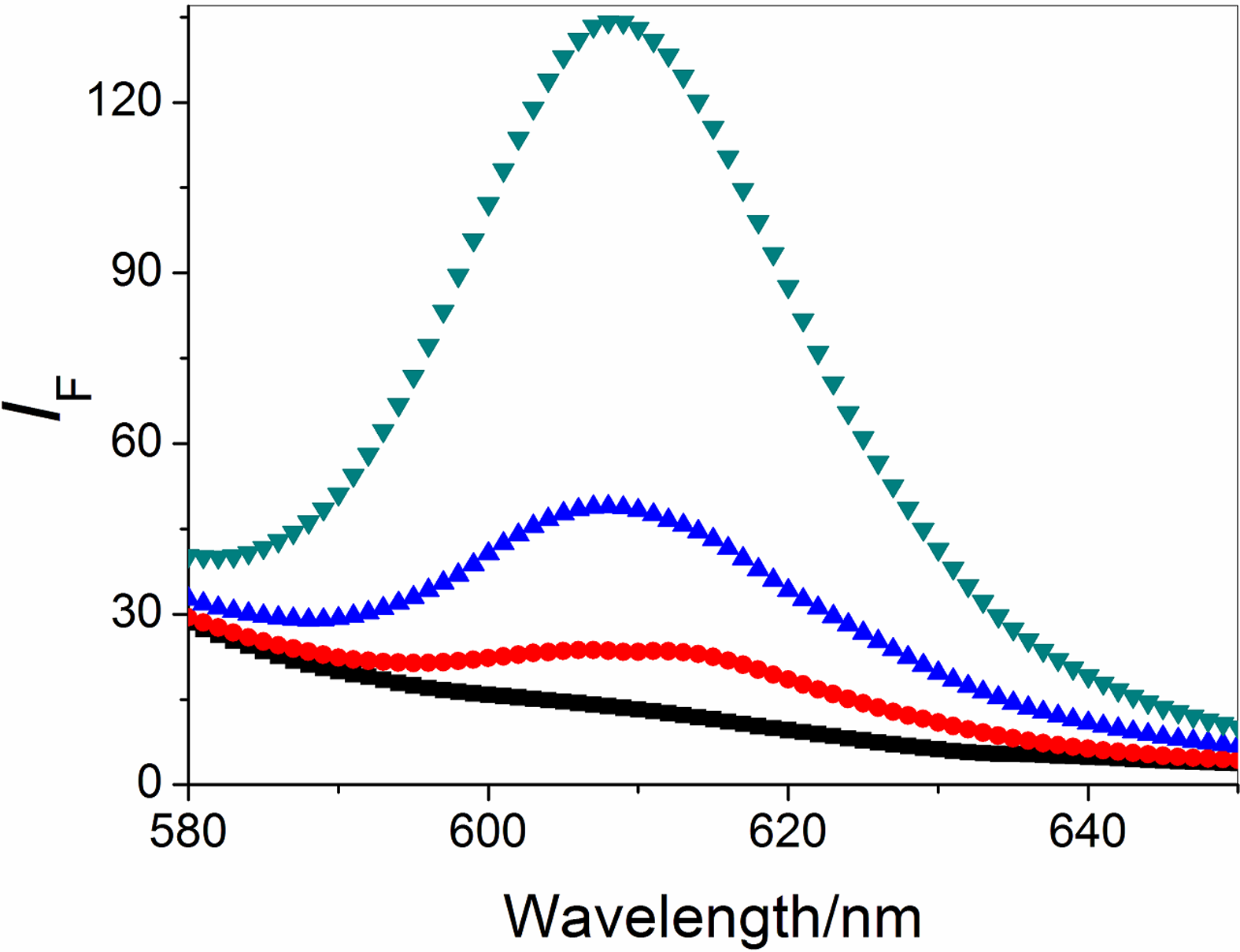

Supplement: Figure S2 — Fluorescence spectra of the supernatant of the cocktail of MMPs-Apt1-PrP-Apt2-QDs separated by magnetic field. PrPRes: 0.00, 3.42, 6.84, 13.86×10−7 mol/L from the bottom up. (TIF) [file pone.0053935.s002.tif]
